# Knockdown of *vps54* aggravates tamoxifen-induced cytotoxicity in fission yeast

**DOI:** 10.5808/gi.21049

**Published:** 2021-12-31

**Authors:** Sol Lee, Miyoung Nam, Ah-Reum Lee, Seung-Tae Baek, Min Jung Kim, Ju Seong Kim, Andrew Hyunsoo Kong, Minho Lee, Sook-Jeong Lee, Seon-Young Kim, Dong-Uk Kim, Kwang-Lae Hoe

**Affiliations:** 1 Department of New Drug Development, Chungnam National University, Daejeon 34134, Korea; 2 Morrissey College of Arts and Sciences, Boston College, Boston 02467, MA, USA; 3 Department of Life Science, Dongguk University-Seoul, Goyang 10326, Korea; 4 Department of Bioactive Material Science, Jeonbuk National University, Jeonju 54896, Korea; 5 Personalized Medicine Research Center, Korea Research Institute of Bioscience & Biotechnology (KRIBB), Daejeon 34141, Korea; 6 Rare Disease Research Center, Korea Research Institute of Bioscience & Biotechnology (KRIBB), Daejeon 34141, Korea; 7 Korea Research Institute of Chemistry & Technology, Daejeon 34141, Korea

**Keywords:** antifungal, estrogen receptor, tamoxifen, vesicle, *vps54*, yeast

## Abstract

Tamoxifen (TAM) is an anticancer drug used to treat estrogen receptor (ER)‒positive breast cancer. However, its ER-independent cytotoxic and antifungal activities have prompted debates on its mechanism of action. To achieve a better understanding of the ER-independent antifungal action mechanisms of TAM, we systematically identified TAM-sensitive genes through microarray screening of the heterozygous gene deletion library in fission yeast (*Schizosaccharomyces pombe*). Secondary confirmation was followed by a spotting assay, finally yielding 13 TAM-sensitive genes under the drug-induced haploinsufficient condition. For these 13 TAM-sensitive genes, we conducted a comparative analysis of their Gene Ontology (GO) ‘biological process’ terms identified from other genome-wide screenings of the budding yeast deletion library and the MCF7 breast cancer cell line. Several TAM-sensitive genes overlapped between the yeast strains and MCF7 in GO terms including ‘cell cycle’ (*cdc2*, *rik1*, *pas1*, and *leo1*), ‘signaling’ (*sck2*, *oga1*, and *cki3*), and ‘vesicle-mediated transport’ (*SPCC126.08c*, *vps54*, *sec72*, and *tvp15*), suggesting their roles in the ER-independent cytotoxic effects of TAM. We recently reported that the *cki3* gene with the ‘signaling’ GO term was related to the ER-independent antifungal action mechanisms of TAM in yeast. In this study, we report that haploinsufficiency of the essential *vps54* gene, which encodes the GARP complex subunit, significantly aggravated TAM sensitivity and led to an enlarged vesicle structure in comparison with the SP286 control strain. These results strongly suggest that the vesicle-mediated transport process might be another action mechanism of the ER-independent antifungal or cytotoxic effects of TAM.

## Introduction

Tamoxifen (TAM) is an estrogen receptor (ER) antagonist used to treat ER-positive breast cancer [1]. Thus, it is classified as a selective ER modulator (SERM). In fact, TAM has diverse effects on eukaryotic cell physiology [2], including modulation of growth signaling [3], regulation of the cell cycle [4], induction of apoptosis [5], modulation of intracellular calcium release [6], antioxidant activity [7], antiangiogenic properties [8], and vesicle-mediated transport [9]. Consistent with its plethora of cellular effects, TAM is also involved in regulating a number of cellular proteins beside ER, including calmodulin, protein kinase C (PKC), [10], phospholipase C [11], phosphoinositide kinase (PIK) [12], and V-ATPase [13].

Despite its categorization as a SERM, TAM also exerts antitumor activity against ER-negative breast cancer [9] and nonmelanoma skin cancer [14]. The ER-independent effects require approximately 10- to 100-fold higher concentrations of TAM than the ER-dependent effects [15]. The ER-independent effects might be partly attributed to the interference of TAM with diverse cellular enzymes, as has been previously reported [10,13]. Moreover, TAM has a narrow spectrum of antifungal activity against several yeast species [16,17] such as *Saccharomyces cerevisiae* (budding yeast) [18,19], *Schizosaccharomyces pombe* (fission yeast) [20], and *Candida albicans* [17]. The phenomena of ER-independent antitumor and antifungal activities have prompted debate on the action mechanism of TAM [2].

Budding and fission yeast species are useful unicellular model organisms [21]. In particular, the development of gene deletion libraries equipped with built-in barcodes in a gene-specific manner has opened the era of parallel analysis to screen for sensitive or resistant genes at a genome-wide scale in response to drugs and chemicals of interest [22] under the principle of drug-induced haploinsufficiency [23]. In this regard, a compendium of TAM-sensitive genes has been constructed through drug-induced haploinsufficiency-based screening of the gene deletion library [18,24] and compared with those identified from the MCF7 breast tumor cell line through knockdown-based (RNAi) genome-wide screening [25]. Comparative research revealed that the effects of TAM were related to several signaling processes in common, including phosphoinositide-dependent kinase 1 (PDK1), PKC, PIK, calmodulin, many growth-related signaling genes and/or oncogenes such as the RAS signaling pathway [25,26].

In this study, we aimed to find a novel mechanism of the ER-independent antifungal effects of TAM, using the fission yeast heterozygous gene deletion library comprising all essential and viable genes [27]. Through a comparison of TAM-sensitive genes between yeast and the MCF7 breast cancer cell line, we found that the modulation of vesicle-mediated transport could be an action mechanism of the ER-independent antifungal activity of TAM in fission yeast.

## Methods

### Chemicals, medium, and the gene deletion library

All chemicals and reagents were obtained from Sigma-Aldrich (St. Louis, MO, USA), unless stated otherwise. Yeast extract and agar were purchased from BD Difco (Sparks, MD, USA). For the systematic screening of TAM-sensitive target genes, we used the heterozygous gene deletion library of fission yeast constructed in a previous study [27]. Briefly, the library represents 98.4% (4,836/4,914) of all protein-coding genes, consisting of 1,260 essential genes and 3,576 non-essential genes. All the deletion strains are available from Bioneer (Daejeon, Korea).

### Half-maximal inhibitory concentration assay

The diploid control SP286 cell (*h*^+^/*h*^+^; *ade*6-M210/*ade*6-M216, *leu*1-32/*leu*1-32, *ura*4-D18/*ura*4-D18) was cultivated to the exponential phase in YES medium (0.5% yeast extract, 3% glucose, and appropriate amino acid supplements) and diluted to an optical density at 600 nm (OD_600_) of 0.05 (~1×10^6^ cells/mL) with the same YES medium. The cells were aliquoted into 96-well plates in triplicate and treated with 5-fold serial concentrations of TAM dissolved in 0.1% DMSO. After cultivating the cells for 17 h at 30ºC, their growth profiles were then measured by OD_600_. The half-maximal inhibitory concentration (IC_50_) value was calculated by a sigmoidal dose-response equation in GraphPad Prism (La Jolla, CA, USA).

### Genome-wide screening of TAM-sensitive genes in fission yeast

The systematic screening of TAM-sensitive genes against 15 µM TAM was performed as previously described [22,27]. Microarray screening was performed using a custom-made GeneChip (48K KRIBB_SP2, Thermo Fisher Scientific, Waltham, MA, USA) and fluorescence-labeled probes were prepared by polymerase chain reaction of the pair of barcodes [27]. TAM-sensitive heterozygous target strains were primarily selected by the criterion of relative growth fitness of <0.92 (p < 0.01) compared with the untreated diploid control strain SP286.

### Spotting assay

The primarily screened TAM-sensitive strains were confirmed using spotting assays based on individual growth fitness. Cells in the log phase were diluted to an OD_600_ = 0.5 in YES medium and spotted in 5-fold serial dilutions onto YES agar plates with or without 65 µM TAM. Compared with the growth fitness of the diploid control strain SP286, the screened TAM-sensitive strains were classified by their degree of sensitivity to TAM as follows: severe (SSS) when growth fitness decreased by more than 2 serial dilutions (>25-fold sensitivity); moderate (SS), between 1 to 2 serial dilutions (5–25-fold sensitivity); and mild (S), less than 1 serial dilution (< 5-fold sensitivity). The relevant TAM-sensitive genes were then subjected to Gene Ontology (GO) analysis using the GO Resource (http://geneontology.org/) and/or the Pombase (https://www.pombase.org/).

### Microscopy of vesicles

Cells were cultivated to the log phase in YES medium at 30°C in the presence of 20 µM TAM in 0.1% DMSO under vigorous aeration conditions. The cells were harvested, resuspended in YES medium, and treated with the FM4-64 staining dye (Thermo Fisher Scientific) to a final concentration of 10 μM at 30°C for 30 min. The cells were washed, resuspended in YES medium, and incubated at 27°C for 1 h. Their vesicles were visualized using a fluorescence microscope (Leica DM5000B, Wetzlar, Germany) equipped with a digital CCD camera (DFC350FX). Differential interference contrast images were used as controls.

### Statistical analysis

All experiments were analyzed using triplicate samples and repeated at least 3 times. Data are presented as the mean ± SD, unless indicated otherwise. Statistical comparisons between groups were performed using the Student t-test. Results with p-values < 0.05 were considered statistically significant.

## Results and Discussion

### Genome-wide screening of TAM-sensitive genes using the fission yeast heterozygous gene deletion library

**Fig. 1.**
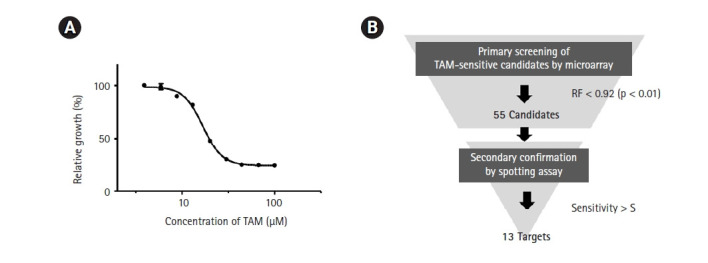
Strategy for genome-wide screening of tamoxifen (TAM)-sensitive heterozygous strains. (A) Measurement of the IC_50_ of TAM. The SP286 diploid control cells were treated with the indicated concentrations of TAM in 1% DMSO. After an additional cultivation for 17 h, the growth fitness was estimated by measuring optical density at 600 nm (OD_600_; n = 3). (B) Schematic drawing of a genome-wide screening of TAM-sensitive target strains. The fission yeast heterozygous deletion library was treated with 15 µM TAM. Primarily, 55 TAM-sensitive candidate strains were selected by the criterion of relative growth fitness (RF) of <0.92 (p < 0.05) compared with the untreated (1% DMSO) SP286 control strain, and subject to a subsequent spotting assay to confirm the candidate strains.

As a first step in genome-wide screening of TAM-sensitive target genes, we determined the IC_50_ of TAM in the SP286 fission yeast diploid strain. According to our previous genome-wide screenings, an optimal concentration of drugs to treat a gene deletion library is lower than the IC_50_. As shown in Fig. 1A, the IC_50_ of TAM was determined to be 17 µM in SP286. Thus, the primary screening was performed with 15 µM TAM. The primary screening and the secondary confirmation processes were performed following the strategy shown in Fig. 1B.

**Fig. 2.**
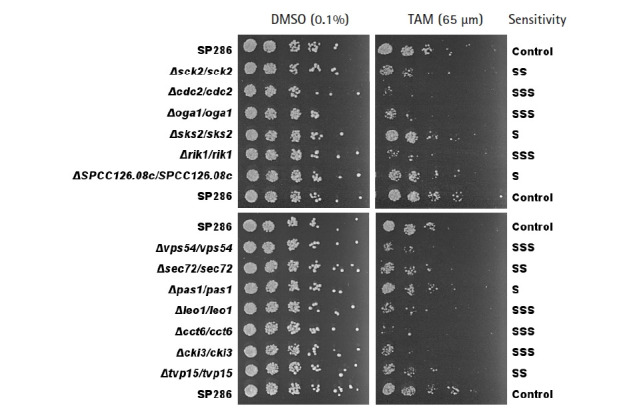
Confirmation of the tamoxifen (TAM)-sensitive candidate strains by spotting assays. TAM-sensitive strains primarily screened by microarray were confirmed by a spotting assay on plates containing 65 µM TAM, compared with the SP286 control strain (on top, middle, and bottom). The cells were 5-fold diluted serially. Their TAM sensitivity was classified as S (mild), SS (moderate), and SSS (severe).

The primary screening yielded 55 candidates (data not shown). The secondary confirmation of the primary candidates by a spotting assay resulted in 13 TAM-sensitive heterozygous strains, compared with the SP286 diploid control strain (Fig. 2). In terms of TAM sensitivity, there were seven severe (SSS), three moderate (SS), and three mild (S) strains. They corresponded to 10 viable (non-essential) and three essential target genes in terms of dispensability. Next, the GO terms of the 13 TAM target genes were examined in terms of biological processes, as shown in Table 1. Their GO terms were related to the following processes: ‘cell cycle’ (*cdc2*, *rik1*, *pas1*, and *leo1*), ‘signaling’ (*sck2*, *oga1*, and *cki3*), ‘vesicle-mediated transport’ (*SPCC126.08c*, *vps54*, *sec72*, and *tvp15*), and ‘protein folding’ (*cct6* and *sks2*).

### Comparison analysis of TAM-sensitive genes revealed that the GO terms related to ‘cell cycle,’ ‘signaling,’ and ‘vesicle-mediated transport’ were shared between the yeasts and MCF7

Upon identifying the 13 TAM-sensitive genes in the study, we compared their GO terms with those of TAM-sensitive genes identified in budding yeast [18] and the MCF7 breast cancer cell line [25].

When the TAM-sensitive genes from fission yeast were compared with those from MCF7 cells and budding yeast, several GO terms overlapped, including ‘cell cycle,’ ‘signaling,’ and ‘vesicle-mediated transport’ (Table 2). As TAM affected growth fitness in both types of yeast, the 3 common GO terms are likely to be related to the ER-independent cytotoxicity of TAM.

Recently, we reported that the knockdown of CSNK1G2, the mammalian orthologous gene of the yeast *cki3* gene associated with the GO term ‘signaling,’ affected cytotoxicity in an ER-dependent or -independent manner in a breast cancer cell line [20]. TAM-induced cytotoxicity is stronger in ER-positive cells than in ER-negative cells, because CSNK1G2 differently modulates the components of the phosphoinositide 3-kinase/AKT/mammalian target of rapamycin/S6K signaling pathway to ERK depending on ER. In yeasts, TAM could induce ER-independent cytotoxicity because TAM modulates the growth-related signaling pathway despite the absence of ER. The TAM-sensitive genes related to the cell cycle clearly appear to be involved with growth fitness in response to TAM. Notably, the association with the GO term ‘vesicle-mediated transport’ was a novel result. This finding is consistent with an accumulating body of evidence suggesting that the integrity of vesicles plays a key role in the cellular transport of chemicals and drugs [30].

### Knockdown of the *vps54* gene aggravates TAM-induced cytotoxicity and disturbs vesicle-mediated structures

The above findings prompted us to examine how the genes classified as being related to vesicle-mediated transport were related to the ER-independent antifungal or cytotoxic effects of TAM in fission yeast. Out of the 4 TAM-sensitive genes classified as related to the GO term ‘vesicle-mediated transport’ (*SPCC126.08c*, *vps54*, *sec72*, and *tvp15*), only the *vps54* gene, which encodes a GARP complex subunit protein, was essential in terms of dispensability. Thus, *vps54* was selected for further experiments, because essential genes are feasible for a functional study.

**Fig. 3.**
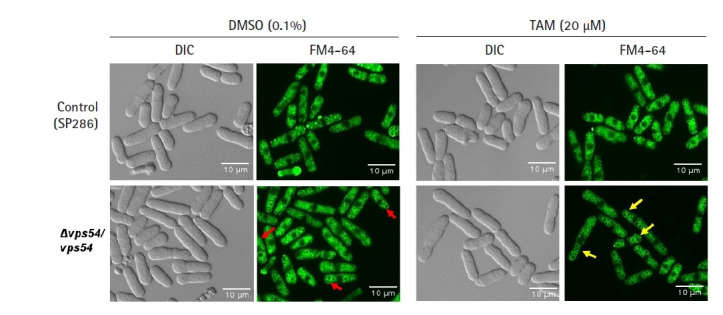
Tamoxifen (TAM)-induced cytotoxicity via enlargement of vesicles. After the *vps54* heterozygous strain was treated with or without 20 µM TAM, its vesicle morphology was visualized by the FM4-64 staining dye and examined using fluorescent microscopy using its differential interference contrast image as a basis, compared with the SP286 control strain. Notably, the *vps54* heterozygous strain showed enlarged vesicles (arrows in red) compared with the SP286 control strain. Moreover, the TAM treatment aggravated the cytotoxicity in the *vps54* heterozygous strain, along with more enlarged vesicles (arrows in yellow) than in the SP286 control strain. DIC, differential interference contrast.

As the *vps* gene family has been reported to affect the integrity of vesicles in terms of their amount and shape [31], we investigated whether its knockdown would affect growth fitness and cause a change in the number or shape of vesicles in response to TAM. Even without TAM treatment, the *vps54* heterozygous mutant showed a high penetrance of enlarged vesicles (red arrows in Fig. 3) without any detectable change in cell shape (Fig. 3) and growth fitness (Fig. 2), compared with the SP286 control strain. The results suggest that 2 copies of the *vps54* gene are required to maintain the stability of vesicle size. When treated with TAM, the *vps54* heterozygous mutants showed more enlarged vesicles (yellow arrows in Fig. 3) along with aggravated cytotoxicity (Fig. 2), compared with the SP286 control. It is likely that haploinsufficiency of *vps54* caused abnormal vesicle shape, leading to TAM-induced cytotoxicity. Consistent with these results, TAM has been reported to affect vesicle-mediated transport in mammalian cells [9], including exocytosis and vesicular release [32].

On the contrary, there is an accumulating body of evidence reporting that blockade of the proton V-ATPase might affect the transport of drugs and metabolites due to malfunctioning vacuolar pH in mammalian cell lines [13,33]. However, this was not the case in fission yeast, as the heterozygous deletion mutants of *vma* genes encoding V-ATPase were not sensitive to TAM treatment (data not shown).

In this study, for the first time in fission yeast, we have found that one of the action mechanisms of the ER-independent antifungal activity of TAM is related to vesicle-mediated transport, as in mammalian cells. Further in-depth research is needed to clarify the details of how TAM aggravates abnormal vesicle structure in the *vps54* heterozygous strain and how abnormal vesicles are related to TAM-induced cytotoxicity.

## Figures and Tables

**Table 1.** List of the 13 TAM-sensitive heterozygous strains

Gene name	Gene description	Biological process	Sensitivity (dispensability)
*cdc2*	Cyclin-dependent protein kinase	Cell cycle [4]	SSS (E)
*pas1*	Cyclin Pas1		S (V)
*rik1*	CLRC ubiquitin ligase complex WD repeat protein		SSS (V)
*leo1*	RNA polymerase II associated Paf1 complex subunit		SSS (V)
*cki3*	Ser/thr protein kinase	Signaling [28]	SSS (V)
*oga1*	Stm1 homolog Oga1		SSS (V)
*sck2*	Ser/thr protein kinase S6K		SS (V)
*sec72*	Arf GEF Sec72	Vesicle-mediated transport [13]	SS (V)
*SPCC126.08c*	Lectin family glycoprotein receptor		S (V)
*tvp15*	COPI-coated vesicle associated protein		SS (V)
*vps54*	GARP complex subunit		SSS (E)
*cct6*	Chaperonin-containing T-complex zeta subunit	Protein folding [29]	SSS (E)
*sks2*	Hsp70 family heat shock protein		S (V)

TAM, tamoxifen; S, mild; SS, moderate; SSS, severe; E, essential; V, viable.

**Table 2.** Comparison of TAM-sensitive genes identified from the fission and budding yeasts and the MCF7 mammalian cell line

GO term: Biological process	Organism (method)		
	Fission yeast (microarray)	Budding yeast (microarray)	MCF7 cell line (RNAi)
Cell cycle	*rik1, cdc2, pas1, leo1*	*AMA1, SHE1, HOG1*	*CIT, PRKCL2, PIM2, PRKCA, ILK, PRKACB, PRKDC, PRKACB*
Signaling	*oga1, sck2, cki3*	*MKK2, INM2*	*PRKCZ, PDK1, KRAS, PPP1R15B, AKT1, PIK3C2B, IRAK3, PIK3C2B, CD3E, RRAS2, GRK7*
Vesicle-mediated transport	*vps54, SPCC126.08c, sec72, tvp15*	*NEO1*	*ABL1, CALM3, TMPRSS2, ACK1, PIP5K1A*
Gene expression	-	*POP4, PRP46, GCD2, RIT1*	*EDF1, IRAK3*
Protein folding	*cct6, sks2*	-	-
Cell redox homeostasis	-	*TRR1, PRX1*	-
Cell differentiation	-	-	*TPM4*
Response to estrogen	-	-	*ESR1*
Miscellaneous	-	*YNL179C, HTC1, NNR2, NOC2, PBA1*	*FLJ23074, C10orf72, C15orf55/NUT*

TAM, tamoxifen; GO, Gene Ontology.
